# YOLOv11-MFF: A multi-scale frequency-adaptive fusion network for enhanced CXR anomaly detection

**DOI:** 10.1371/journal.pone.0334283

**Published:** 2025-10-24

**Authors:** Li Guan, Ruting Zhang, Yi Zhao

**Affiliations:** 1 Department of Smart Manufacturing, Industrial Perception and Intelligent Manufacturing Equipment Engineering Research Center of Jiangsu Province, Nanjing Vocational University of Industry Technology, Nanjing, Jiangsu, China; 2 Department of Radiotherapy, The First Affiliated Hospital of Soochow University, Suzhou, Jiangsu, China; 3 Mechanical and Electronic Systems Engineering Course, Graduate School of Engineering, Takushoku University, Tokyo, Japan; University of Maryland College Park, UNITED STATES OF AMERICA

## Abstract

Chest X-ray (CXR) represents one of the most widely utilized clinical diagnostic tools for thoracic diseases. Nevertheless, computer-aided diagnosis based on chest radiographs still faces considerable challenges in anomaly detection. Certain lesions in CXRs exhibit subtle radiographic characteristics with ambiguous boundaries, low pixel occupancy, and weak contrast. While existing studies primarily focus on improving multi-scale feature fusion, they frequently overlook complications arising from background noise and varied lesion morphology. This study introduces YOLOv11-MFF, an enhanced YOLOv11 network with three key innovations. Specifically, a novel Frequency-Adaptive Hybrid Gate (FAHG) is developed to improve contrast differentiation between lesions and background. A Multi Scale Parallel Large Convolution (MSPLC) block is designed and integrated with the original C3k2 module to expand receptive fields and enhance long-range modeling capacity. Furthermore, a Feature Fusion module (FF) is introduced to reinforce target-relevant feature representation through channel-wise modulation via weight recalibration mechanisms. Benefiting from these advancements, the network achieves significant improvements in detecting multi-scale and overlapping lesions. Experimental results on the public VinDr-CXR dataset demonstrate that YOLOv11-MFF outperforms state-of-the-art models, achieving a precision of 48.2%, recall of 42.5%, mAP@0.5 of 41.5%, and mAP@0.5:0.95 of 22.6%.

## 1. Introduction

Chest X-ray (CXR) technology has become the most frequent and cost-effective examination method for thoracic abnormalities in medical facilities [1]. Its low cost and operational ease facilitate accessibility in underdeveloped regions, contributing to the vast number of CXRs generated worldwide. The substantial volume of CXRs requiring individual interpretation by radiologists has considerably increased their workload, demanding significant time and effort. Computer-Aided Diagnostic (CAD) systems are expected to effectively assist radiologists by automatically identifying and localizing abnormalities in CXRs [2]. With recent advances in computing power and the availability of large amounts of datasets, deep learning techniques have flourished over the past few years [3]. Numerous deep-learning-based methods have been integrated into CAD systems. Previous studies have established that neural networks, particularly Convolutional Neural Networks (CNNs), can aid radiologists in interpreting CXRs and reducing misdiagnosis rates.

Most models employed in CAD systems within previous studies are based on supervised learning and classification networks. For instance, several deep learning approaches [4–6] were developed and verified on private datasets for binary classification of CXRs. However, the ability to identify multiple abnormalities would be more advantageous for radiologists. Recent efforts have targeted the detection of specific chest diseases, such as lung cancer [7], nodules [8,9], penumothorax [10], tuberculosis [11], or pneumonia [12]. Nonetheless, clinical CXR images often present multiple co-existing abnormalities. Hence, a model designed for a specific chest disease may offer limited assistance in radiologists’ interpretations. Other studies [13–15] proposed CNN-based classification models for multi-abnormality detection, developed and evaluated on public CXR datasets (ChestX-ray14 dataset). However, these models can only provide final classification results per CXR without localization information.

For object detection techniques in computer vision, a high-quality CXR dataset containing both category and location labels (bounding boxes) is essential to identify and locate multiple abnormalities. Yet, most existing CXR datasets include only abnormality labels, restricting the advancement of machine learning algorithms in CAD systems. To address this issue, Nguyen [16] introduced the VinDr-CXR dataset in 2022, comprising CXR images in DICOM format along with publicly available location annotations for abnormalities. Several studies [17,18] developed models based on this dataset, with one network, CXR-RefineDet, achieving 16.9% mAP@50 and 6.8 fps. Moreover, this dataset was used in a 2021 Kaggle competition, where the winning model reached a mAP@50 of 32.4%. The current state-of-the-art performance (33.8% mAP@50) was attained in 2024 by S. N. Hao [19] using an enhanced YOLOv8-based architecture.

However, contemporary research predominantly emphasizes multi-scale feature fusion improvements while overlooking interference from background noise. In chest radiographs, some small lesions exhibit semi-transparent properties, ill-defined boundaries, low pixel occupancy, and weak contrast, potentially leading to missed detection or misclassification. This study proposes a YOLOv11-based CXR anomaly detection method capable of localizing and identifying 14 distinct pathological types. Key innovations include:

1. **Frequency-Adaptive Hybrid Gate (FAHG)**: Utilizing Fast Fourier Transform (FFT), this component decomposes feature maps into low-, medium-, and high-frequency components from a frequency-domain perspective. It selectively amplifies low- and medium-frequency features — rich in structural and textural information — while suppressing high-frequency components containing edge noise, thereby enhancing target-background contrast.
2. **Multi-Scale Parallel Large Convolution Block (MSPLC)**: Integrated into the original C3k2 module, this structure incorporates parallel multi-scale dilated convolution layers and aggregates their outputs. The design enlarges receptive fields, improves long-range modeling, and captures multi-scale image characteristics to enhance accuracy in complex scenarios.
3. **Feature Fusion Module (FF)**: By employing weight recalibration mechanisms, this module performs channel-wise modulation on concatenated cross-scale features. It preserves global semantic information from deep layers (for large lesion localization) while strengthening detail retention in shallow layers (suppressing interference from ribs and vascular structures), collectively improving noise robustness and detection precision.

The paper is organized as follows: Section 2 reviews state-of-the-art research in CXR anomaly detection. Section 3 elaborates on the improved model architecture and its advantages. Section 4 presents experimental design and results analysis. Section 5 discusses limitations. Sections 6 and 7 provide extended discussions and conclusions, respectively. A graphical abstract illustrating the study concept and key findings is available as S1 File.

## 2. Related works

Among various deep learning algorithms, the YOLO (You Only Look Once) series [20] has gained popularity in real-time detection tasks due to its simplicity and adaptability to resource-constrained environments. YOLO-based algorithms have been widely applied in domains such as autonomous driving, security surveillance, and medical imaging, demonstrating notable versatility and efficiency.

In CXR anomaly detection, many researchers have also investigated YOLO-based enhancements. Cho [21] combined DenseNet principles with the YOLOv2 framework, achieving high detection rates for five disease categories on a small private dataset. However, limited by the dataset’s scale and narrow disease scope, further validation is necessary to confirm its efficacy. Hao [19] proposed YOLO-CXR, a multi-disease detection network based on YOLOv8, which replaces standard convolutional layers with RefConv layers to improve feature extraction. It also incorporates an Efficient Channel and Local Attention (ECLA) mechanism to enhance spatial sensitivity and introduces a dedicated small-lesion detection head along with Selective Feature Fusion (SFF). Evaluated on the VinDr-CXR dataset, it achieved state-of-the-art results with 0.338 mAP@0.5, 0.167 mAP@0.5:0.95, and 0.365 recall.

In comparison, YOLOv11 represents a more recent version of object detection networks. Zhao [22] developed a lightweight FASPA fast pyramid attention mechanism within the YOLOv11 architecture, replacing the C3k2 module with FASPA to significantly reduce network parameters while maintaining detection performance. On the MIMIC Chest X-ray dataset, the model attained 94.1% mAP@0.5. However, it is limited to binary classification of pneumonia signs and lacks generalization for multi-class detection involving complex thoracic abnormalities such as pneumothorax, pleural effusion, and pulmonary nodules.

**Fig 1:**
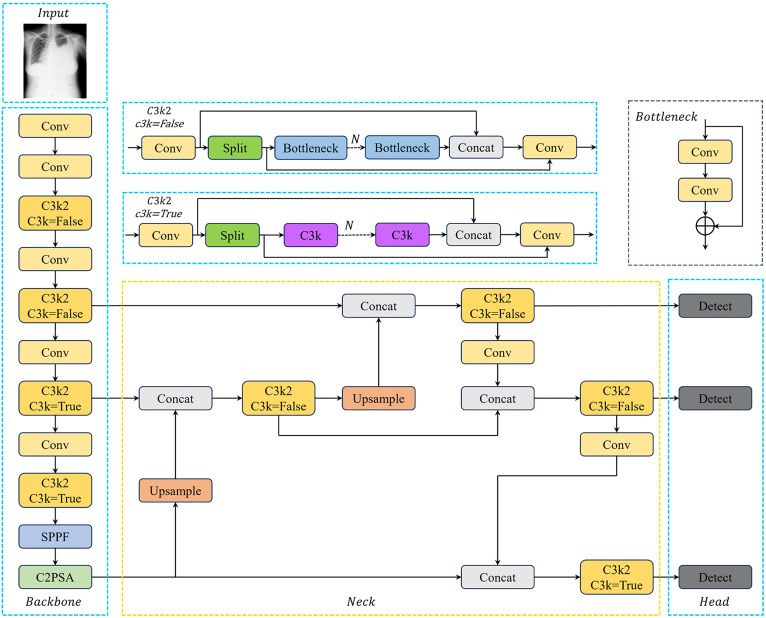
The structure of YOLOv11.

As illustrated in Fig 1, YOLOv11's input module integrates advanced data augmentation techniques, including mosaic and mixup, building upon YOLOv8's adaptive anchor box computation. By adopting an anchor-free approach, YOLOv11 achieves flexible adaptation to various input resolutions without explicit anchor box calculations.

Compared to YOLOv8, key innovations involve replacing C2f modules in the backbone and neck with the C3k2 module for finer-grained feature extraction and appending the C2PSA module after SPPF to enhance spatial attention mechanisms. The framework also implements distributed focal loss to optimize bounding box prediction. On the VinDr-CXR dataset, YOLOv11 achieves 42.2% precision, 38.7% recall, 38.8% mAP@0.5, and 21.4% mAP@0.5:0.95.

## 3. The proposed YOLOv11-MFF model

This section outlines the network architecture of YOLOv11-MFF, analyzes the operational mechanisms of the MSPLC, FF, and FAHG modules, and illustrates their impacts on anomaly detection through integrated visualization heatmaps.

### 3.1. Network structure

Although YOLOv11 has shown promising performance across various object detection tasks, it encounters specific challenges in chest radiograph anomaly recognition:

1. Multi-scale object coexistence and overlap in abnormal CXRs (e.g., simultaneous presence of micronodules and extensive pulmonary consolidation), where YOLOv11's receptive field is insufficient for precise multi-scale lesion localization
2. Complex lesion morphologies with subtle contrast between pathological and normal tissues, rendering conventional YOLOv11 less effective in capturing faint and low-contrast targets.

To address these limitations, we propose YOLOv11-MFF, an enhanced detection framework incorporating three core innovations. The model introduces a Multi-Scale Parallel Large Convolution (MSPLC) module integrated with the original C3k2 module. This architecture enhances receptive fields and long-range modeling via stacked parallel multi-scale dilated convolutional layers and feature aggregation, effectively capturing multi-scale image characteristics. Additionally, a Feature Fusion (FF) module performs channel-wise modulation on concatenated cross-scale features through weight recalibration. This design preserves global semantic information from deep features (aiding large lesion localization) while enhancing detail retention in shallow features (suppressing interference from ribs and vessels).

**Fig 2:**
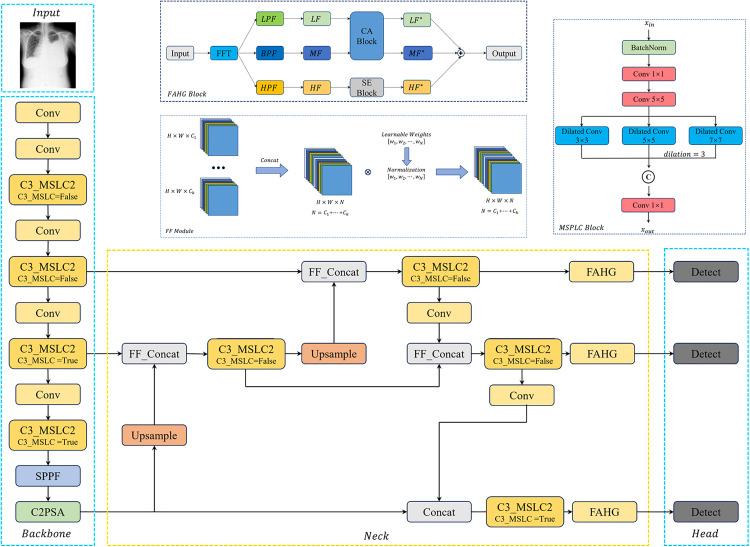
The structure of YOLOv11-MFF.

Moreover, a Frequency-Adaptive Hybrid Gate (FAHG) module leverages Fast Fourier Transform to decompose feature maps into low-, medium-, and high-frequency components. This frequency-aware processing amplifies structurally informative low and medium frequencies while suppressing noise-prone high frequencies, thereby improving target-background contrast. The overall architecture of YOLOv11-MFF is depicted in Fig 2.

### 3.2. MSPLC block

**Fig 3:**
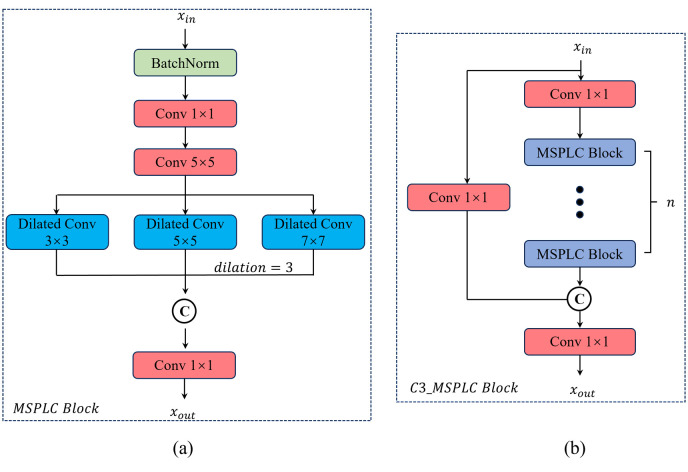
The structure of the MSPLC block: (a) Overall architecture of MSPLC block; (b) The structure of the C3_MSPLC block (integrates the MSPLC block into the C3k block.).

To enhance the model's receptive field and long-range modeling capabilities, we propose a Multi-Scale Parallel Large Convolution Block (MSPLC) that integrates dilated convolutions [23] and multi-branch feature concatenation [24] to capture multi-scale characteristics of images. The module’s structure is illustrated in Fig 3a.

In the MSPLC block, the input feature map *X* undergoes feature extraction through a *BN* layer followed by two convolutional layers. Both convolutional layers use a stride of 1 and maintain the original channel count. The computational procedure is formalized in Equation (1), where BN(·) denotes the batch normalization, ${Conv}_{i\times j}$ represents convolution with an *i* × *j* kernel, and *Y* is the output feature map.


$$Y={Conv}_{\text{5}\times \text{5}}({Conv}_{\text{1}\times \text{1}}(BN(X)))$$ (1)

The MSPLC block then applies three parallel large-kernel dilated convolutions with kernel sizes of 3 × 3, 5 × 5, and 7 × 7, all with a dilation rate of 3 and stride of 1. This configuration enables cross-scale pathological feature capture while maintaining real-time processing. Specifically: The 7 × 7 dilated convolution targets holistic cardiopulmonary contour anomalies (e.g., lobar infiltration patterns). The 5 × 5 pathway detects meso-scale structural abnormalities such as rib fractures and pleural thickening. The 3 × 3 pathway captures micronodules and textural details. The computational processes are formalized in Equations (2)–(4), where ${Dilated\_Conv}_{i\times j}$ denotes the dilated convolution with kernel size *i* × *j*, and $Y_{i\times j}$ is the corresponding output.


$$Y_{\text{3}\times \text{3}}={Dilated\_Conv}_{\text{3}\times \text{3}}(X)$$ (2)

$$Y_{\text{5}\times \text{5}}={Dilated\_Conv}_{\text{5}\times \text{5}}(X)$$ (3)

$$Y_{\text{7}\times \text{7}}={Dilated\_Conv}_{\text{7}\times \text{7}}(X)$$ (4)

The multi-branch feature maps are concatenated via Concat(·) and then processed by a 1 × 1 convolutional layer for dimensionality reduction, ensuring the output channel count matches the input. This is expressed in Equation (5):


$$Y={Conv}_{\text{1}\times \text{1}}(Concat(Y_{\text{3}\times \text{3}},Y_{\text{5}\times \text{5}},Y_{\text{7}\times \text{7}}))$$ (5)

This architecture facilitates multi-scale information fusion, creating a lesion size-adaptive dynamic receptive field that significantly improves anomaly detection in complex clinical scenarios such as overlapping pathologies.

Integration of the “C3_MSPLC” module into YOLOv11 is illustrated in Fig 3b. This enhanced module replaces the original “C3K” component within the “C3K2” backbone via a Boolean switch: when “C3_MSPLC=False”, the standard “C3K” is replaced with the base MSPLC; when “C3_MSPLC=True”, the full “C3_MSPLC” is activated. This preserves YOLOv11’s native topology while adding adaptive multi-scale processing.

**Fig 4:**
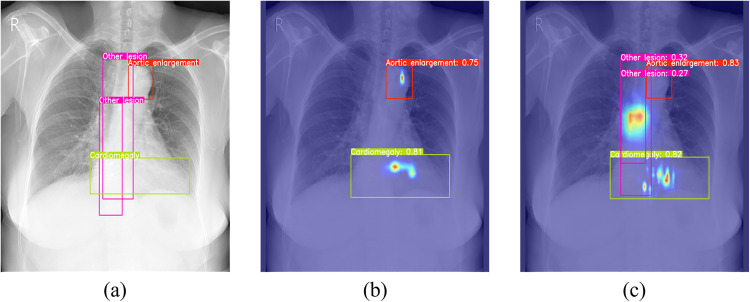
Heatmap comparison: (a) groundtruth; (b)YOLOv11n; (c)YOLOv11n-MSPLC.

To illustrate receptive field differences, heatmap visualizations compare YOLOv11n and YOLOv11n-MSPLC (Fig 4). The heatmaps overlay the model’s attention distribution onto original radiographs, with color intensity indicating attention strength. Comparative analysis shows that YOLOv11n-MSPLC outperforms YOLOv11n in global contextual understanding through cross-scale feature fusion, exhibiting more comprehensive lesion coverage and anatomical relevance.

### 3.3. FF module

**Fig 5:**
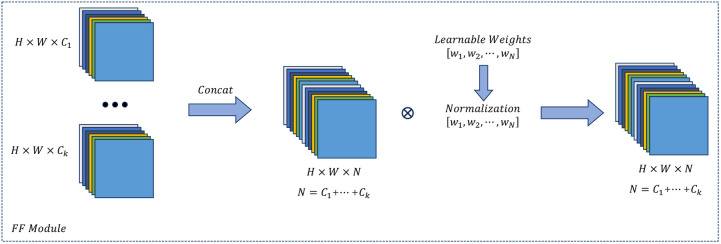
The structure of the FF module.

YOLOv11 conventionally uses equal-weighted concatenation for channel feature fusion, which fails to account for contribution disparities among multi-level feature maps. To address this, we implement a Feature Fusion Module [25] (FF Module) as a structural replacement, detailed in Fig 5.

The FF Module incorporates learnable weight vectors to dynamically assign channel-wise importance based on chest lesion characteristics, including morphological traits (e.g., ill-defined margins of ground-glass opacities, high-contrast textures of calcified nodules) and scale distributions (small lesions prone to feature dilution in deep layers). This adaptive mechanism prioritizes task-relevant channel information during fusion, specifically enhancing edge responses of small lesions in shallow features. Additionally, to handle multi-scale coexistence (e.g., concurrent micronodules and consolidations), the FF Module performs channel-wise modulation on concatenated cross-scale features via weight recalibration. This design preserves global semantic information in deep features (for large lesion localization) while amplifying detail retention in shallow features (suppressing background interference).

Denote the input feature map as $X\in R^{B\times C\times H\times W}$ with learnable parameters $w\in R^{C}$. The computation is formalized in Equations (6) and (7):


$$\alpha =softmax(w)\in R^{C}$$ (6)

$$Y=X\cdot \alpha^{\left(\text{1},C,\text{1},\text{1}\right)}\in R^{B\times C\times H\times W}$$ (7)

**Fig 6:**
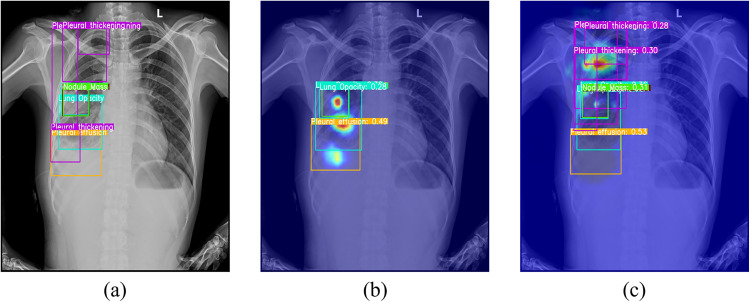
Heatmap comparison: (a) groundtruth; (b)YOLOv11n; (c)YOLOv11n-FF.

Fig 6 demonstrates the feature extraction differences between YOLOv11n and its FF module-enhanced variant YOLOv11n-FF. The comparison shows that YOLOv11-FF uses dynamic channel weighting to amplify textural details in shallow features and counteract information dilution in deep features from downsampling, thereby strengthening activation in pathological regions.

### 3.4. FAHG module

**Fig 7:**
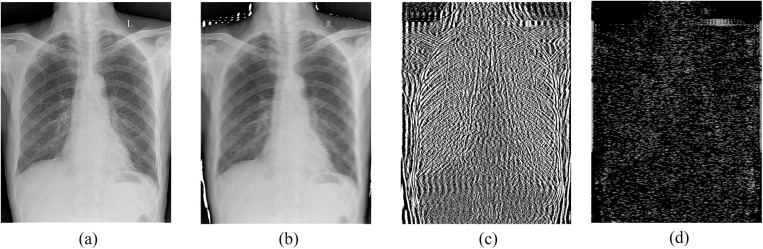
Frequency domain decomposition of CXR: (a) Original CXR; (b) Low-frequency component (anatomical structures); (c) Mid-frequency component (tissue textures); (d) High-frequency component (pathological details and edges).

In medical imaging, anatomical structures and pathological lesions exhibit heterogeneous manifestations corresponding to distinct frequency characteristics. Using raw chest radiographs as an example, Fourier transform decomposition yields low-, medium-, and high-frequency components, visualized in Fig 7.

**Fig 8:**
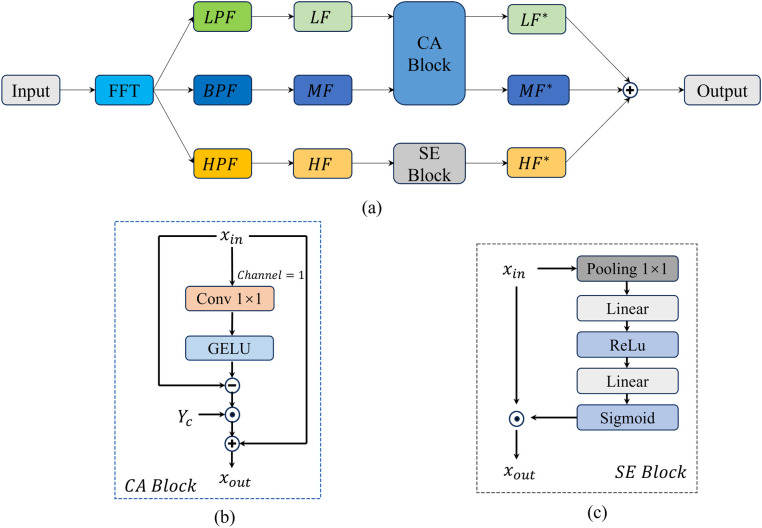
The structure of the FAHG module: (a) Overall architecture of FAHG module; (b) CA Block; (c) SE Block.

Generally, low-frequency components convey global contour information (e.g., organ shapes and positions); mid-frequency components contain structural and textural information for distinguishing tissue boundaries; high-frequency components relate to edges and noise [26]. Theoretically, enhancing low and mid frequencies while selectively amplifying high-frequency signals to highlight edges and suppress noise can improve feature map contrast. Accordingly, this paper proposes a Frequency-Adaptive Hybrid Gate (FAHG), shown in Fig 8a.

In the FAHG module, the input feature map is first transformed to the frequency domain via Fast Fourier Transform (FFT), as in Equation (8).


$$F(u,v)=\sum_{x=\text{0}}^{M-\text{1}}\sum_{y=\text{0}}^{N-\text{1}}f(x,y)\cdot e^{-i\text{2}\pi \left(\frac{ux}{M}+\frac{vy}{N}\right)}$$ (8)

Here, *F*(*u, v*) is the frequency-domain complex matrix, with frequency variables *u* and *v* in the ranges 0≤u≤M and 0≤v≤N. *f* (*x*, *y*) is the spatial-domain image, with coordinates (*x*, *y*) and ranges 0≤x≤M, 0≤y≤N. *M* and *N* are the image height and width, and *i* is the imaginary unit.

Different frequency components are extracted using low-pass, mid-pass, and high-pass filters, as in Equations (9)–(11).


$$F_{low}(u,v)=F(u,v)\cdot H_{low\_mask}(u,v)$$ (9)

$$F_{mid}(u,v)=F(u,v)\cdot H_{mid\_mask}(u,v)$$ (10)

$$F_{high}(u,v)=F(u,v)\cdot H_{high\_mask}(u,v)$$ (11)

Here, $H_{low\_mask}(u,v)$ is a low-pass filter retaining low-frequency components (1 when $\sqrt{u^{\text{2}}+v^{\text{2}}}\leq D_{\text{0}}$, otherwise 0). $H_{mid\_mask}(u,v)$ is a mid-pass filter (1 when $D_{\text{0}}<\sqrt{u^{\text{2}}+v^{\text{2}}}\leq D_{\text{1}}$ otherwise 0). $H_{high\_mask}(u,v)$ is a high-pass filter (1 when $\sqrt{u^{\text{2}}+v^{\text{2}}}>D_{\text{1}}$, otherwise 0). *D*_0_ and *D*_1_ are predefined thresholds, configurable as learnable parameters.

Filtered frequency-domain signals are converted back to the spatial domain via inverse FFT, as in Equations (12)–(14).


$$f_{low}(x,y)=\frac{\text{1}}{MN}\sum_{u=\text{0}}^{M-\text{1}}\sum_{v=\text{0}}^{N-\text{1}}F_{low}(u,v)\cdot e^{i\text{2}\pi \left(\frac{ux}{M}+\frac{vy}{N}\right)}$$ (12)

$$f_{mid}(x,y)=\frac{\text{1}}{MN}\sum_{u=\text{0}}^{M-\text{1}}\sum_{v=\text{0}}^{N-\text{1}}F_{mid}(u,v)\cdot e^{i\text{2}\pi \left(\frac{ux}{M}+\frac{vy}{N}\right)}$$ (13)

$$f_{high}(x,y)=\frac{\text{1}}{MN}\sum_{u=\text{0}}^{M-\text{1}}\sum_{v=\text{0}}^{N-\text{1}}F_{high}(u,v)\cdot e^{i\text{2}\pi \left(\frac{ux}{M}+\frac{vy}{N}\right)}$$ (14)

Here, $f_{low}(x,y)$, $f_{mid}(x,y)$, and $f_{high}(x,y)$ are spatial-domain feature maps of low-, mid-, and high-frequency components, corresponding to *LF*, *MF* and *HF* in Fig 8a.

To address distinct physical meanings and task requirements across frequency bands, the CA (Channel Aggregation) module (Fig 8b) and SE (Squeeze-and-Excitation) module (Fig 8c) are applied differentially. Low- and mid-frequency components carry core semantic information (e.g., organ shapes, tissue boundaries) but may have redundant or weakly correlated features across channels. The CA module dynamically fuses complementary cross-channel information to strengthen global consistency of low-frequency features and multi-scale structural representations of mid-frequency features. In contrast, high-frequency components contain fine edges and noise, with significant SNR variations across channels. The SE module adaptively recalibrates channel weights to suppress noise-dominated channels and amplify those with valid high-frequency features (e.g., subtle lesion edges), avoiding noise diffusion and enabling efficient refinement.

For the CA module, its computation is formulated in Equation (15).


$$CA(X)=X+\gamma_{C}⊙\left(X-GELU({Conv}_{\text{1}\times \text{1}}(X))\right)$$ (15)

Here, *CA*(*X*) is the output, *X* is the input, $\gamma_{C}$ is a learnable channel scaling factor, GELU(·) is the activation function, and ${Conv}_{\text{1}\times \text{1}}(\cdot )$ is used for dimensionality reduction. If *X* has dimensions [B,C,H,W], $\gamma_{C}$ is sized [1,C,1,1], and ${Conv}_{\text{1}\times \text{1}}(X)$ outputs [B,1,H,W].

**Fig 9:**
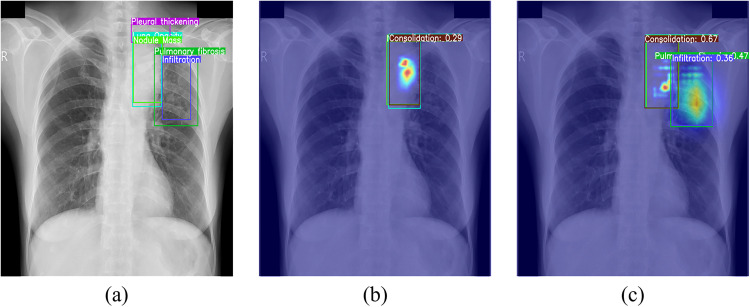
Heatmap comparison: (a) groundtruth; (b) YOLOv11n; (c) YOLOv11n-FAHG.

To intuitively demonstrate the practical effects of the FAHG module in frequency-specific processing, heatmap visualizations are compared between YOLOv11n and the FAHG-enhanced variant YOLOv11n-FAHG. As shown in Fig 9, YOLOv11n-FAHG, due to Channel Aggregation’s enhancement of low-frequency global contours (e.g., cardiopulmonary boundaries) and mid-frequency structural textures (e.g., rib gaps, abnormal calcifications), effectively reduces the impact of background interference and high-frequency noise on detection results.

## 4. Experiments

This section details the evaluation metrics, hardware configurations, hyperparameters, and dataset composition. The superiority of YOLOv11-MFF is validated through ablation studies.

### 4.1. Model evaluation metrics

Common object detection metrics include Precision (P), Recall (R), Average Precision (AP), and Mean Average Precision (mAP). P measures prediction accuracy: the proportion of correct predictions among positive predictions. R measures recall: the proportion of positive samples correctly predicted. AP measures detection accuracy for a specific category, defined as the area under the PR curve at a specific IoU threshold. mAP averages AP across all categories. Formulas are given in Equations (16)–(19):


$$P=\frac{TP}{TP+FP}\times \text{100}\%$$ (16)

$$R=\frac{TP}{TP+FN}\times \text{100}\%$$ (17)

$$AP=\;\int_{\text{0}}^{\text{1}}P(R)dR$$ (18)

$$MAP=\;\frac{\text{1}}{n}\sum_{i=\text{1}}^{n}{AP}_{i}$$ (19)

Here, TP (True Positive) is the number of correctly predicted positive samples, FP (False Positive) is incorrectly predicted positives, FN (False Negative) is incorrectly predicted negatives, and n represents the total number of categories.

### 4.2. Experimental parameters

Experimental parameters include hardware specifications and algorithmic variables. To ensure consistency, all algorithms were evaluated under identical system configurations. Training used uniform parameters across approaches.

Hardware comprised a 13th Gen Intel Core i7-13700KF CPU, NVIDIA GeForce RTX 4080 GPU (16GB VRAM), and 48GB RAM. The software environment was Windows 11 ProPlus, PyTorch 2.0.1, Python 3.10.11, and Anaconda 23.3.1.

### 4.3. Hyperparameter settings

The model used Stochastic Gradient Descent (SGD) with a learning rate of 0.01 and momentum of 0.937. Training ran for 300 epochs with a batch size of 16. A three-phase warmup gradually increased momentum from 0.8. The loss function combined bounding box localization loss (7.5×), object classification loss (0.5×), and distribution-focused regularization (1.5×).

### 4.4. Data exploration

The VinDr-CXR dataset comprises 15,000 CXRs in DICOM format, collected from two major Vietnamese healthcare institutions: 108 Military Central Hospital and Hanoi Medical University Hospital. This dataset (publicly available at https://vindr.ai/datasets/cxr) contains a total of 22,719 professionally annotated bounding boxes encompassing 14 thoracic abnormality types. Each image was independently annotated by three board-certified radiologists to ensure diagnostic consistency.

This study adopted a random partitioning strategy (with random seed set to 1234) to divide the dataset into training, validation, and test sets, with the specific sample distribution shown in Table 1.

**Table 1:** Detailed statistics of the dataset after preprocessing.

Split	Number of Images	Number of Bounding Boxes	Percentage %
Traing set	12000	18200	80
Validation set	2250	3407	15
Test set	750	1112	5
Total	15000	22719	100

**Fig 10:**
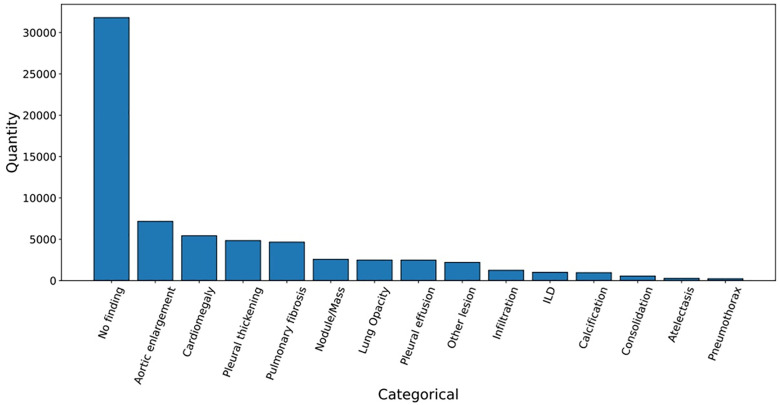
Distribution of abnormalities on the VinDr-CXR dataset.

**Fig 11:**
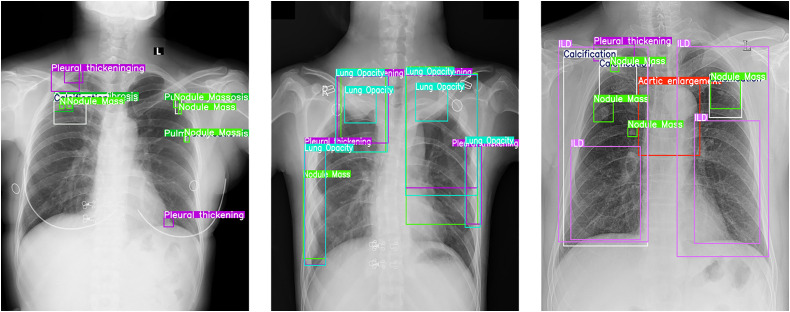
CXRs with annotations.

Fig 10 shows a long-tail label distribution, with normal CXRs predominant. Underrepresented abnormalities include Pneumothorax, Atelectasis, Consolidation, and Calcification. To mitigate class imbalance, we excluded normal radiographs and used class-weighted resampling. Fig 11 presents representative examples of radiographic manifestations for all 14 annotated pathological categories within the dataset.

**Fig 12:**
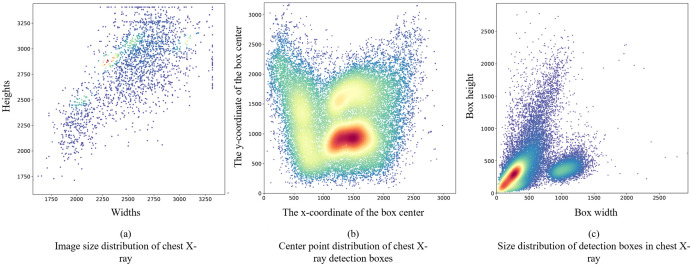
Distribution of abnormal bounding box and CXR resolution.

Statistical analyses covered image resolution, bounding box centroids, and sizes (Fig 12a–12c). A systematic analysis of lesion dimensions across abnormality categories revealed significant prevalence of small targets (following established criteria [27,28]) in three pathological manifestations: pulmonary nodules/masses demonstrated the highest proportion (39.69%), followed by calcifications (17.67%) and miscellaneous lesions (10.26%).

### 4.5. Data enhancement

Generally, the distribution of abnormal categories in CXR datasets is not balanced. For example, in VinDr-CXR, Pneumothorax, Atelectasis, Consolidation, and Calculation are underrepresented, while Aortic enlargement and Cardiomegaly are overrepresented. To alleviate the impact of these imbalances during training, this work adopts an upsampling method based on weighted data. The specific steps are as follows:

1. Get the number of occurrences of each category in the training set and calculate the weight of each category based on Equation (20).

$${WC}_{i}=\frac{\sum_{i=\text{1}}^{T}O_{i}}{O_{i}}$$ (20)

Here, *WC*_*i*_ represents the weight of the i-th category. T represents the number of categories. *O*_*i*_ represents the number of occurrences of the i-th category.

1. Calculate the weight of each data in the training set and normalize it based on Equations (21)–(23).

$${WS}_{i}=Agg\_fun({Bbox}_{\text{1}},{Bbox}_{\text{2}},\cdots ,{Bbox}_{n})$$ (21)

$${Bbox}_{j}={WC}_{k}$$ (22)

$${n\_WS}_{i}=\frac{{WS}_{i}}{\sum_{l=\text{1}}^{h}{WS}_{l}}$$ (23)

Here, *WS*_*i*_ represents the weight of the i-th data. n represents the number of bounding boxes contained in the i-th data. *Bbox*_*j*_ represents the weight of the j-th bounding box. Agg_fun(·) represents the aggregation function. k represents the category to which the abnormality in the j-th bounding box belongs. ${n\_WS}_{i}$ represents the weight of the i-th data after normalization. *h* represents the amount of data in the training set.

1. Repeated sampling is performed with the data weight as the corresponding probability when loading data during training.

Since the weight of each category is inversely proportional to the number of occurrences, the weight of the less represented category is greater. This indirectly increases the probability of sampling CXRs containing underrepresented categories, making such samples more likely to be repeated.

**Fig 13:**
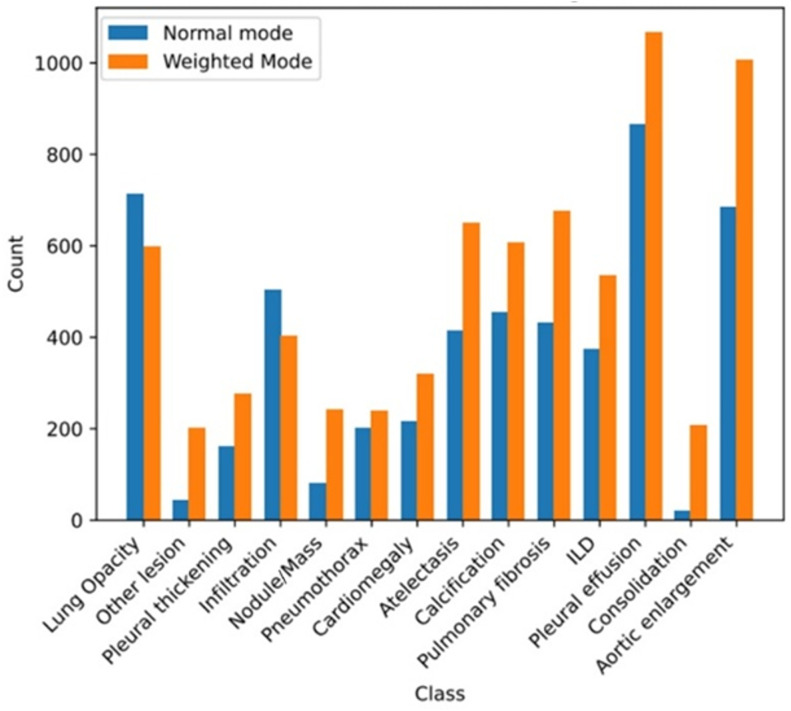
Comparison of category distribution before and after implementing data-weighted upsampling.

This work adopts sum function as the Agg_fun(·). Fig 13 shows the comparison of category distribution before and after implementing weighted upsampling. It can be seen from Fig 13 that weighted upsampling can alleviate the imbalanced distribution of categories.

### 4.6. Experiment results and analysis

**Fig 14:**
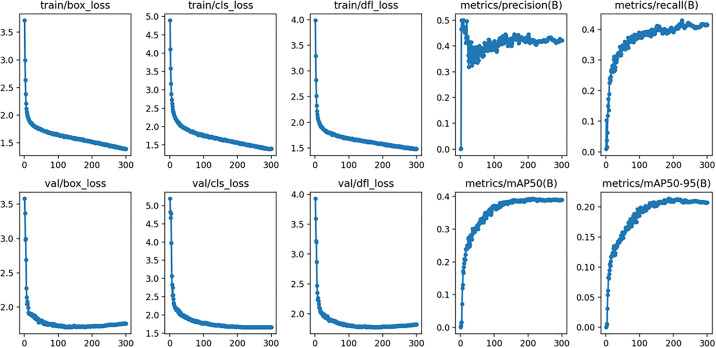
Training and validation losses and metric progression.

To evaluate the performance of the YOLOv11n-MFF model, tests were conducted on the dataset described in Section 4.4. After 300 training epochs, the model achieved convergence. As shown in Fig 14: the bounding box loss (box_loss) measures the deviation between predicted and ground-truth boxes, where smaller values indicate higher detection precision; the classification loss (cls_loss) evaluates the discrepancy between predicted and true categories, with lower values reflecting superior classification accuracy; the distribution focal loss (dfl_loss) achieves finer coordinate localization by transforming continuous coordinate predictions into discrete probability distributions, where smaller values denote improved prediction precision.

**Fig 15:**
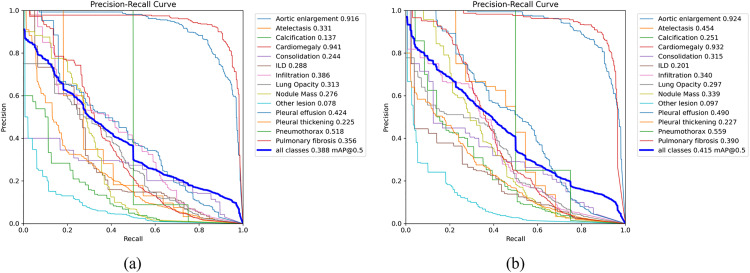
Precision-recall curve: (a) YOLOv11n; (b)YOLOv11n-MFF.

As illustrated in Fig 15, compared to the baseline YOLOv11n model, YOLOv11n-MFF demonstrates significant performance gains in detecting thoracic abnormalities. Specifically, YOLOv11n-MFF improves detection accuracy for 10 out of 12 anomaly categories compared to YOLOv11n. Notably, the “Calcification” and “Atelectasis” categories exhibit remarkable improvements of 83.2% and 37.1%, respectively. However, slight declines are observed for “ILD” and “Infiltration” categories. Evaluated by the mAP@0.5 metric, the overall detection accuracy across all categories improves by 7.0%.

Furthermore, the proposed model is benchmarked against mainstream architectures including YOLOv5nu, YOLOv8n, YOLOv10n, and YOLOv11s, with its superiority validated across four evaluation metrics.

To further evaluate model performance, each predicted bounding box in the dataset was classified as a positive or negative sample using the following procedure: first, among the unmatched ground truth boxes, the ground truth box most closely matching the current predicted box (i.e., of the same category and with the highest IoU) was identified; second, whether the IoU between the predicted box and this ground truth box exceeded the threshold (0.5) was determined. If the threshold was exceeded, the ground truth box was considered successfully matched, the sample was labeled as positive, and its confidence score and category were recorded; otherwise (if no match was found or the IoU was insufficient), it was classified as a negative sample, with its confidence score and category also recorded. On this basis, ROC curves and the AUC metric were introduced for a more comprehensive evaluation of model performance.

**Fig 16:**
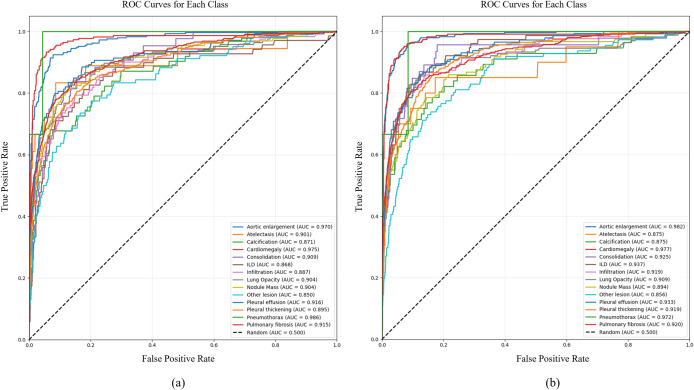
ROC curve: (a) YOLOv11n; (b)YOLOv11n-MFF.

The ROC curves and AUC values for each target category are shown in Fig 16. The YOLOv11n model achieved a macro-average AUC of 0.90, while the YOLOv11n-MFF model reached a macro-average AUC of 0.92, representing an increase of 2.2%. These results indicate that the proposed model performs marginally superior to the baseline model in terms of classification metrics.

**Fig 17:**
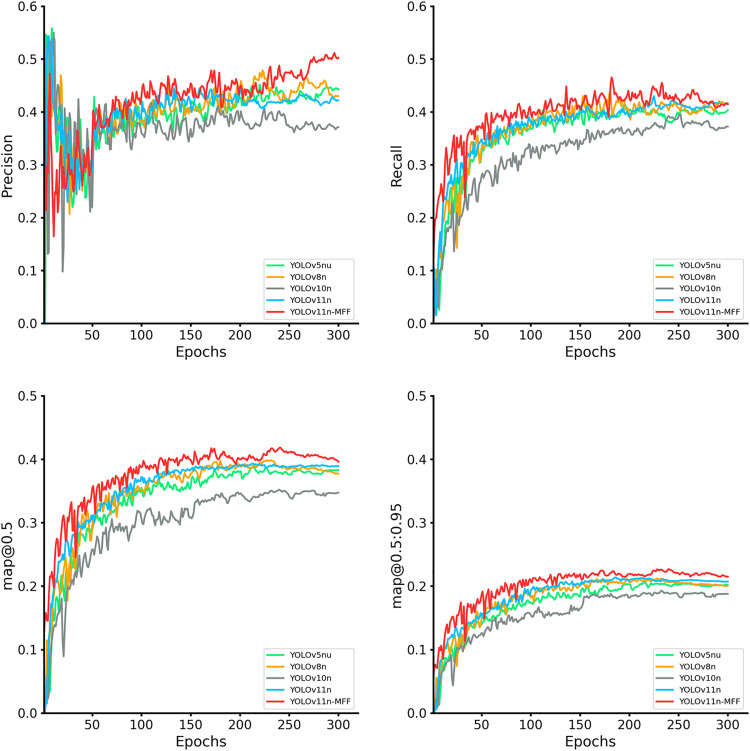
Evaluation and comparison of metric curves of YOLOv11n-MFF and baseline models.

As depicted in Fig 17, YOLOv11n-MFF outperforms other models across all four metrics during most training phases. YOLOv5nu, YOLOv8n, and YOLOv11n exhibit similar performance curves, converging to comparable values in later stages but remaining inferior to YOLOv11n-MFF. YOLOv10n underperforms across most training phases, emerging as the weakest model in this experiment, with significant gaps in precision and mAP@0.5. Under the stricter mAP@0.5:0.95 metric, YOLOv11n-MFF also demonstrates superior detection capabilities.

Notably, this dataset was previously used in a 2021 Kaggle competition, where the winning model achieved 32.40 mAP@50. The current state-of-the-art model proposed by S. N. Hao [19] in 2024, an improved YOLOv8 variant with the data augmentation described in Section 4.5, attains 39.8 mAP@50, 0.217 mAP@0.5:0.95, and 0.395 recall on the test set. In comparison, our experiments achieve 41.5 mAP@50, 0.226 mAP@0.5:0.95, and 0.425 recall, further highlighting the superiority of the proposed YOLOv11-MFF model.

### 4.7. Ablation experiments

To validate the contributions of integrating the MSPLC, FF, and FAHG modules to YOLOv11n-MFF’s performance, ablation experiments are conducted. The training performance of YOLOv11n, YOLOv11n-MSPLC, YOLOv11n-FF, YOLOv11n-FAHG, YOLOv11n-MSPLC+FF, YOLOv11n-MSPLC+FAHG, YOLOv11n-FF+FAHG, and YOLOv11n-MFF is compared across four metrics.

**Fig 18:**
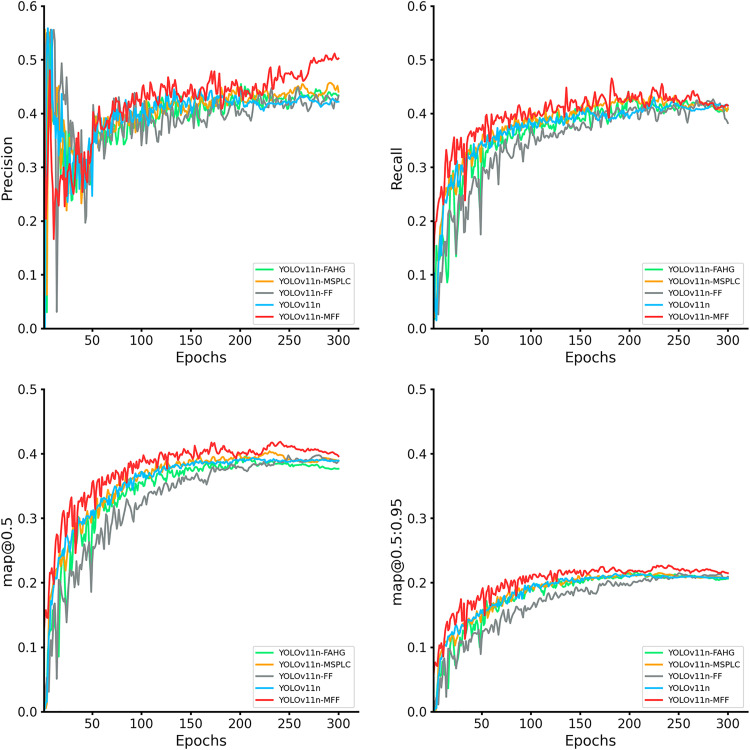
Evaluation and comparison of metric curves of YOLOv11n-MFF and single-module models.

As shown in Fig 18, models with individual modules (FF, MSPLC, or FAHG) exhibit modest improvements over the baseline YOLOv11n across all metrics. YOLOv11n-MSPLC and YOLOv11n-FAHG achieve comparable precision, recall, and mAP@0.5, while YOLOv11n-FF slightly lags in recall. Crucially, YOLOv11n-MFF demonstrates strong performance in precision and mAP@0.5.

When MSPLC, FF, and FAHG modules are jointly integrated to form YOLOv11n-MFF, its precision curve leads throughout most training phases, achieving the highest final value with enhanced stability and convergence in later stages. In recall, YOLOv11n-MFF also outperforms counterparts in most phases. Under both mAP@0.5 and the stricter mAP@0.5:0.95 metrics, YOLOv11n-MFF consistently surpasses all comparative models.

To verify the necessity of combining all three modules, performance differences among YOLOv11n, YOLOv11n-MSPLC+FF, YOLOv11n-MSPLC+FAHG, YOLOv11n-FF+FAHG, and YOLOv11n-MFF are analyzed.

**Fig 19:**
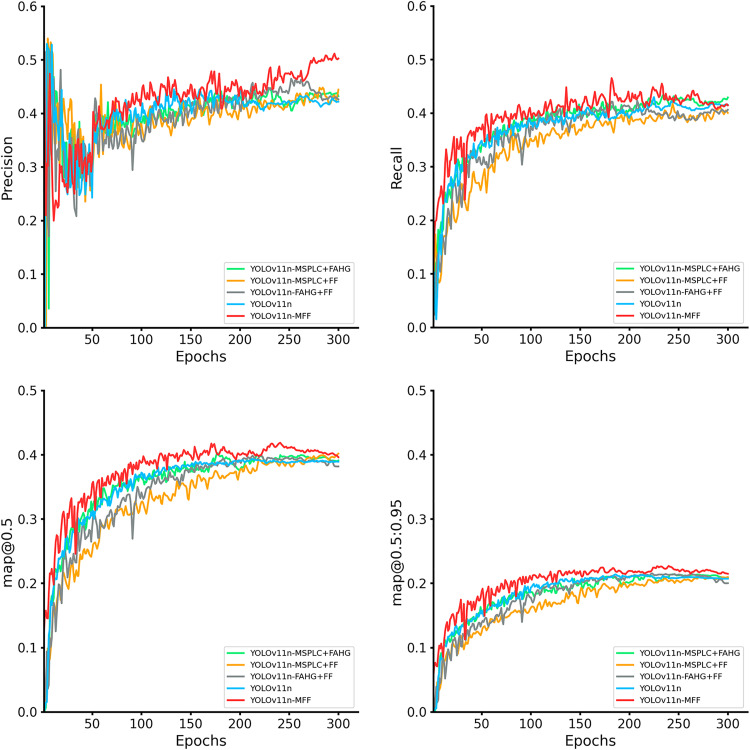
Evaluation and comparison of metric curves of YOLOv11n-MFF and dual-module models.

As shown in Fig 19, models with any two modules generally outperform the baseline across most metrics. However, YOLOv11n-MSPLC+FF matches the baseline in precision and slightly underperforms in mAP@0.5:0.95. In contrast, the full YOLOv11n-MFF model surpasses all dual-module combinations, with leading precision and recall curves, peak final values, and enhanced stability. Under both mAP@0.5 and mAP@0.5:0.95 metrics, YOLOv11n-MFF demonstrates comprehensive superiority, validating its exceptional detection capabilities.

As shown in Table 2, the FF module, with its lightweight design, introduces only minimal parameters and negligible computational overhead, yet increases mAP@0.5 from 38.8 to 39.4. The FAHG module adds 0.1M parameters and 0.2G FLOPs of computational cost while raising mAP@0.5 to 39.5. The MSPLC module contributes an increase of 0.2M parameters and 1.5G FLOPs, along with an improvement in mAP@0.5 to 39.3. After integrating all three modules, YOLOv11n-MFF outperforms all comparative models in precision, recall, mAP@0.5, mAP@0.5:0.95, and F1-score. Compared to the baseline YOLOv11n, it achieves a 14.2% increase in precision, a 9.8% improvement in recall, a 7.0% gain in mAP@0.5, a 5.6% rise in mAP@0.5:0.95, and an 11.8% enhancement in F1-score. Combined with the 2.2% improvement in macro-average AUC shown in Fig 16, these results clearly demonstrate the performance enhancement brought by the module innovations. Meanwhile, the number of parameters increases from 2.6M to 3.1M, and computational complexity rises from 6.4G FLOPs to 9.9G FLOPs. Additionally, YOLOv11n, YOLOv8n, and YOLOv5nu achieve comparable mAP@0.5 results, all outperforming YOLOv10n. In terms of precision, recall, and mAP@0.5:0.95, YOLOv11n-FAHG+FF performs the best among all dual-module configurations.

**Table 2:** Evaluation metric comparison table of YOLOv11n-MFF and other models.

Model	P/%	R/%	map@0.5	map@0.5:0.95	F1	Params/M	FLOPs/G
YOLOv5nu	43.2	40.1	38.8	20.7	41.6	1.8	4.2
YOLOv8n	43.7	42.4	38.9	21.2	43.0	3.0	13.2
YOLOv10n	39.7	37.2	35.2	19.4	38.4	2.7	6.7
YOLOv11n	42.2	38.7	38.8	21.4	40.4	2.6	6.4
YOLOv11n-MSPLC	43.9	40.5	39.3	21.5	42.1	2.8	7.9
YOLOv11n-FAHG	42.9	42.2	39.5	21.6	42.5	2.7	6.5
YOLOv11n-FF	43.5	39.2	39.4	21.5	41.2	2.6	6.4
YOLOv11n-MSPLC+FAHG	43.3	41.1	39.9	21.4	42.2	2.9	8.0
YOLOv11n-MSPLC+FF	42.2	39.6	39.6	21.3	40.9	2.9	8.1
YOLOv11n-FAHG+FF	44.2	42.6	39.9	21.7	43.4	2.7	6.5
YOLOv11n-MFF	**48.2**	**42.5**	**41.5**	**22.6**	**45.2**	3.1	9.9

Compared to the baseline YOLOv11n, YOLOv11n-MFF improves precision by 14.2%, recall by 9.8%, mAP@0.5 by 7.0%, and mAP@0.5:0.95 by 5.6%, robustly validating the performance breakthroughs enabled by module innovations.

**Fig 20:**
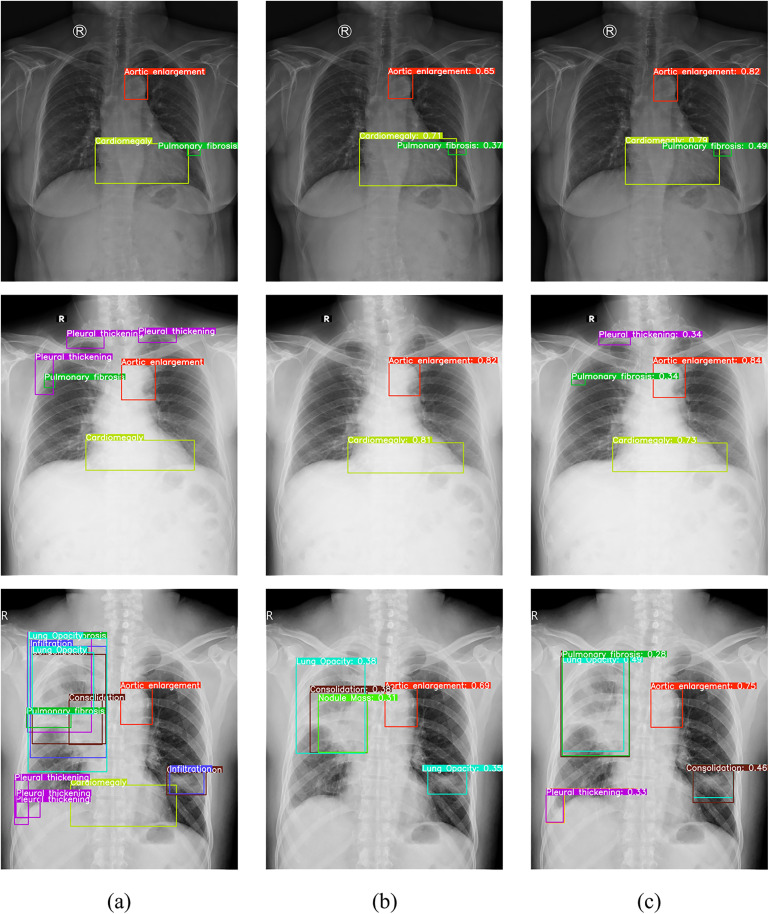
Example of detection result comparison: (a) original image with annotations; (b) YOLOv11n; (c) YOLOv11n-MFF.

Fig 20 visualizes test-set comparisons between YOLOv11n and YOLOv11n-MFF. Results confirm that YOLOv11n-MFF achieves higher classification accuracy and superior recall for complex CXR samples compared to YOLOv11n.

### 4.8. Efficiency analysis

The inference efficiency and computational resource consumption of the model are critical for its practical deployment in clinical settings. A comparison of the inference time and GPU memory consumption for the YOLOv11-MFF model and its individual components is presented in Table 3.

**Table 3:** Comparison of inference efficiency and resource consumption.

Model/Component	Inference Time (ms)	GPU Memory (MB, single image)	Params/M	FLOPs/G
YOLOv11n (Baseline)	8.0	769.6	2.6	6.4
+ MSPLC	+2.9	+40.8	2.8	7.9
+ FF	+0.8	+11.6	2.6	6.4
+ FAHG	+0.9	+14.4	2.7	6.5
YOLOv11n-MFF(Ours)	12.8	950.8	3.1	9.9

It can be observed from Table 3 that the baseline model YOLOv11n achieves an inference time of 7.0 ms per image (equivalent to 143 FPS) on the current hardware. The three newly introduced modules contribute an additional overhead of 4.8 milliseconds: among them, the MSPLC block, due to its parallel large-kernel dilated convolutions, constitutes the most computationally intensive component, adding 2.9 ms of latency; the FAHG module, although involving frequency-domain operations, benefits from highly optimized FFT implementations on modern GPUs and increases inference time by only 0.9 ms; the FF module is the most lightweight, adding only 0.8 ms.

In terms of memory consumption, the complete YOLOv11-MFF model requires 950.8 MB of GPU memory during inference, which can be readily accommodated by most modern GPUs. Compared to the baseline YOLOv11n, it improves precision by 14.2%, recall by 9.8%, mAP@0.5 by 7.0%, mAP@0.5:0.95 by 5.6%, and F1-score by 11.8%, at the cost of a 60.0% increase in inference time and a 23.5% rise in memory usage—a trade-off that is considered acceptable in the field of medical image diagnosis, where accuracy is often the primary concern. Furthermore, the overall inference speed of 12.8 ms (78 FPS) still far exceeds the real-time requirements for clinical deployment.

### 4.9. External validation

External validation of medical AI models is a critical step for assessing their generalization capability and clinical utility, playing a significant role in ensuring robustness across different devices, populations, and medical institutions.

The “Shenzhen Hospital CXR” (abbreviated as SZ-HospCXR) dataset was jointly developed by the U.S. National Library of Medicine, the Third People’s Hospital of Shenzhen, and Guangdong Medical University in China. It is a digital imaging resource focused on tuberculosis diagnosis. This dataset exhibits significant differences from the primary training set used in this study (VinDr-CXR) in terms of patient demographics, imaging equipment, and acquisition parameters. It includes 336 tuberculosis-positive CXRs and 326 normal cases. Images are stored in PNG format with a resolution of approximately 3000 × 3000 pixels and are annotated with mask labels covering 19 abnormality types such as contraction, calcified nodule, miliary lesions, lymph node enlargement, apical thickening, and interlobar fissure thickening.

To adapt this dataset for the validation needs of this study, the following preprocessing steps were applied: first, mask annotations were converted into bounding box format and adjusted to YOLO data specifications; second, based on medical definitions, the original labels in SZ-HospCXR were mapped and integrated into the label system used in this study. For example, the “Calcification” category in this study includes calcified foci, calcified nodules, and calcified lymph nodes excluding those classified separately as nodules and lymph nodes. Therefore, the three corresponding label types from SZ-HospCXR were merged into the “Calcification” category. After this conversion, 10 abnormality categories shared with the model used in this study were consolidated from the dataset, including Atelectasis and Calcification, among others.

The results of testing on this external validation set for the aforementioned 10 common categories are shown in Table 4. It can be observed that for most targets, the performance of the YOLOv11-MFF model experienced a certain degree of decline compared to the internal test set, which is primarily due to differences in data distribution and falls within expected outcomes.

**Table 4:** Model performance on internal and external validation datasets across common findings.

Model Output	Corresponding Label in SZ-HospCXR	No. of Instances (SZ-HospCXR)	AP % (VinDr-CXR)	AP% (SZ-HospCXR)
Atelectasis	Retraction	11	45.4	41.6
Calcification	Calcification (other than nodule & lymphnod), Calcified Nodule, Calcified lymph node	21	25.1	23.9
Consolidation	Severe Infiltrate (Consolidation)	35	31.5	32.2
ILD	Linear Density, Miliary	144	20.1	18.0
Infiltration	Moderate_Infiltrate_(non-linear), Small_Infiltrate_(non-linear)	310	34.0	32.0
Lung Opacity	Cavity	45	29.7	26.8
Nodule/Mass	Calcified Nodule; Clustered Nodule (2 mm-5 mm); Single Nodule (non-calcified)	355	33.9	32.0
Other lesion	Adenopathy; Other; Unknown	53	9.7	12.5
Pleural effusion	Pleural Effusion	59	49.0	44.0
Pleural thickening	Apical Thickening; Pleural Thickening (non-apical); Thickening of interlobar fissure	121	22.7	26.1
Mean AP (mAP@Common)			30.1	28.9

Furthermore, the performance comparison between the YOLOv11-MFF model and baseline models on the SZ-HospCXR dataset is shown in Table 5. It can be seen that although the detection accuracy of the YOLOv11-MFF model on the external validation set decreased compared to the internal test set, its performance still significantly outperforms other comparative models (such as YOLOv11n's 26.2%), further demonstrating that the model proposed in this study possesses strong generalizability and robustness.

**Table 5:** Evaluation of the model’s predictive performance on common classes based on the external validation set (SZ-HospCXR).

Model	Precision%	Recall%	map@0.5	map@0.5:0.95	F1	Macro- AUC
YOLOv8n	32.2	33.1	24.8	13.7	32.6	0.81
YOLOv11n	33.6	32.4	26.2	14.3	33.0	0.84
**YOLOv11-MFF(Ours)**	**38.9**	**33.2**	**28.9**	**15.0**	**35.8**	**0.85**

## 5. Analysis of limitations

Section 4 validated YOLOv11n-MFF’s performance advantages. This section provides qualitative comparisons of correct detections, misses, and false alarms across models. Additionally, YOLOv11n-MFF was tested on noisy CXRs to assess robustness.

### 5.1. Qualitative analysis

**Fig 21:**
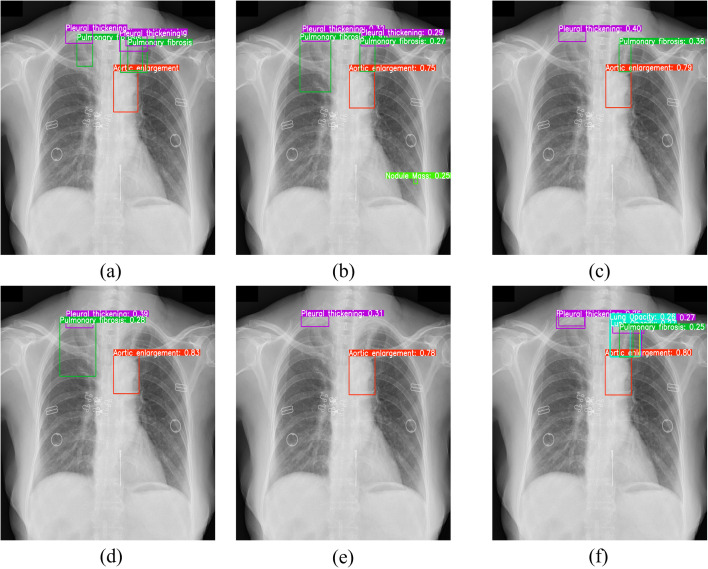
Visual comparison of detection results from different models: (a)original image with annotations; (b)YOLOv5nu; (c)YOLOv8n; (d)YOLOv10n; (e)YOLOv11n; (f)YOLOv11n-MFF.

Fig 21a shows a CXR with three pleural thickening instances, three pulmonary fibrosis lesions, and one aortic enlargement. Fig 21b–21f display results from YOLOv5nu, YOLOv8n, YOLOv10n, YOLOv11n, and YOLOv11n-MFF. All models detected high-contrast, distinct targets well but varied on multi-scale overlapping abnormalities.

Fig 21b–21f displays detection results from YOLOv5nu, YOLOv8n, YOLOv10n, YOLOv11n, and YOLOv11n-MFF. Experiments show that all models detected high-contrast, distinct targets well but varied on multi-scale overlapping abnormalities.

All models detected the central aortic enlargement. YOLOv10n and YOLOv11n missed upper-right pulmonary fibrosis. YOLOv5nu, YOLOv8n, and YOLOv11n missed upper-left pulmonary fibrosis. YOLOv5nu had two false positives upper-right. YOLOv11n-MFF had no false positives or misses.

However, when abnormalities had low contrast and were obscured by rib shadows, YOLOv11n-MFF still produced false detections.

### 5.2. Noise resistance capability analysis

**Fig 22:**
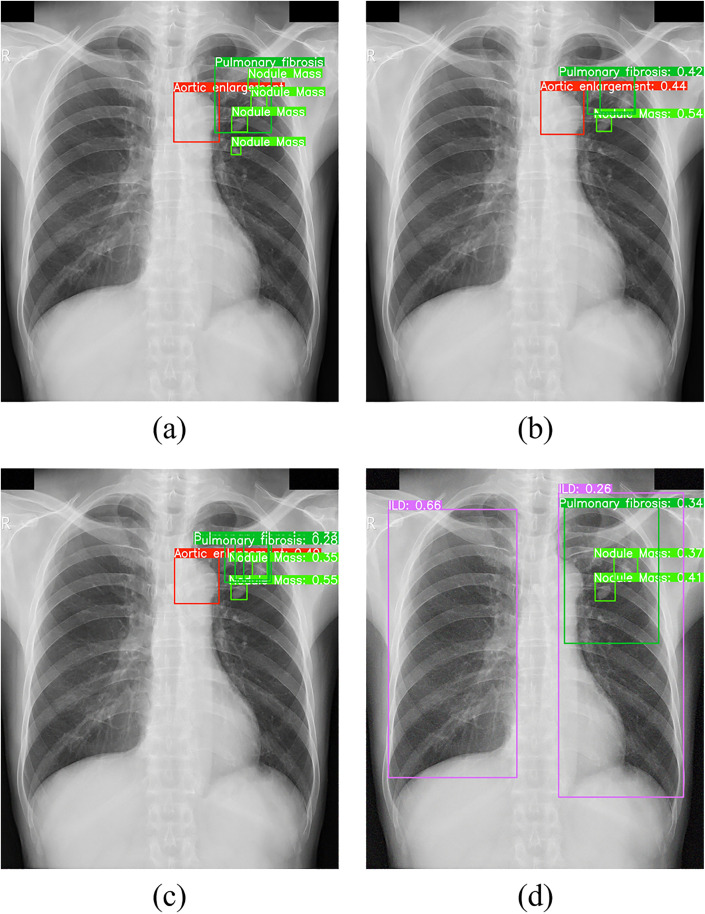
Detection results of YOLOv11n-MFF on with different levels of noise: (a) original image with annotations; (b) without additional noise; (c) with low-intensity noise; (d) with moderate-intensity noise.

To thoroughly evaluate YOLOv11n-MFF's performance on high-noise CXRs, we tested the model on the CXR shown in Fig 22a, containing four nodule masses, one aortic enlargement, and one pulmonary fibrosis. By adding low- and moderate-intensity Gaussian noise to simulate interference, detection results were compared across three scenarios: without additional noise, low-intensity noise, and moderate-intensity noise.

As shown in Fig 22b, YOLOv11n-MFF correctly detects three targets (including one small target) when no additional noise is applied. With low-intensity Gaussian noise added (Fig 22c), the model still detects and correctly identifies four targets, though confidence scores for most detections decrease. The nodule mass missed in Fig 22b and 22c, located in the upper-right region of the CXR, exhibits blurred intensity boundaries that make recognition challenging. When moderate-intensity Gaussian noise is introduced, YOLOv11n-MFF essentially loses its target detection capability.

Experimental results demonstrate that while YOLOv11n-MFF possesses basic noise resistance, it exhibits significant limitations in processing high-noise CXR images. Especially when targets have extremely small sizes and extremely low feature contrast, noise interference drastically weakens the model's effective detection capability.

## 6. Discussion

YOLOv11-MFF shows superior accuracy and noise resistance over YOLOv11 without greatly increasing network depth or parameters. However, missed detections and misclassifications persist, potentially due to: Some lesions appearing as small cavities or cysts, requiring multimodal confirmation (MRI, DSA) for subtyping, leading to annotation discrepancies among radiologists and challenging ground truth. Dataset imbalance—despite augmentation, rare classes like ILD remain underrepresented, limiting feature learning.

CXR annotation requires medical expertise and anonymization, resulting in high costs and scarce public datasets, which primarily constrains model performance.

## 7. Conclusion

To address multi-scale lesion overlaps, diverse morphologies, and low lesion-background contrast, this study proposed YOLOv11-MFF, an enhanced YOLOv11-based network. Experiments demonstrate:

1. The FAHG module effectively amplifies low- and mid-frequency features (rich in structural/textural information) while suppressing high-frequency noise, enhancing contrast and noise resistance.
2. The MSPLC module, integrated into C3k2, uses parallel multi-scale dilated convolutions with aggregation to expand receptive fields and long-range modeling, capturing multi-scale characteristics.
3. The FF module performs channel-wise modulation on cross-scale features via weight recalibration, preserving deep-layer semantic information (for large lesions) while enhancing shallow-layer details (suppressing ribs and vessels). Combined with MSPLC, it enables precise multi-scale localization.

Ablation studies and comparisons with YOLO variants using Precision, Recall, mAP@0.5, and mAP@0.5:0.95 metrics confirm high generalizability, robustness, and accuracy. However, performance gaps remain for small cystic/cavitary lesions, indicating future work directions.

## Supporting information

S1 Fig Graphical abstract. (TIF)

S1 File CLAIM checklist. (PDF)
