# Supplementary figures and images for: YOLOv11-MFF: A multi-scale frequency-adaptive fusion network for enhanced CXR anomaly detection
Source: PLoS One. 2025 Oct 24;20(10):e0334283. doi: 10.1371/journal.pone.0334283 (PMC12551852; doi:10.1371/journal.pone.0334283)

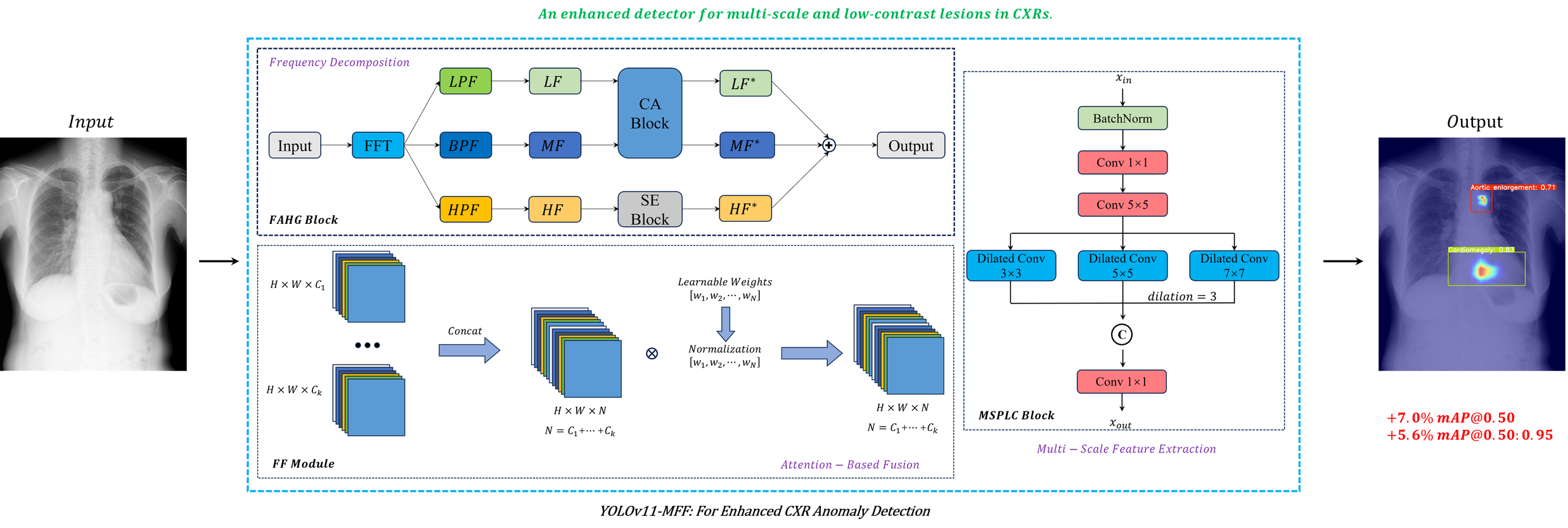

Supplement: S1 Fig — (TIF) [file pone.0334283.s001.tif]
