# Supplementary material for: YOLOv11-MFF: A multi-scale frequency-adaptive fusion network for enhanced CXR anomaly detection
Source: PLoS One. 2025 Oct 24;20(10):e0334283. doi: 10.1371/journal.pone.0334283 (PMC12551852; doi:10.1371/journal.pone.0334283)
Supplement: S1 File — (PDF) [file pone.0334283.s002.pdf]

Checklist for Artificial Intelligence in Medical Imaging (CLAIM)

Section / Topic	No.	Item	Page / Line	No	NA
**TITLE / ABSTRACT**					
	**1**	Identification as a study of AI methodology, specifying the category of technology used (e.g., deep learning)	Title; Abstract	√	
**ABSTRACT**					
	**2**	Summary of study design, methods, results, and conclusions	Abstract	√	
**INTRODUCTION**					
	**3**	Scientific and/or clinical background, including the intended use and role of the AI approach	Sec 1, p.2-4	√	
	**4**	Study aims, objectives, and hypotheses	Sec 1, p.2(end)	√	
**METHODS**					
Study Design	**5**	Prospective or retrospective study	Sec 2, p.4-5	√	
	**6**	Study goal	Sec 1, p.3; Sec 3.1, p.5	√	
Data	**7**	Data sources	Sec 4.4, p.12	√	
	**8**	Inclusion and exclusion criteria	Sec 4.4, p.12	√	
	**9**	Data pre-processing	Sec 4.4, p.13	√	
	**10**	Selection of data subsets	Sec 4.4, p.12	√	
	**11**	De-identification methods			√
	**12**	How missing data were handled	Sec 4.4, p.12	√	
	**13**	Image acquisition protocol			√
Reference Standard	**14**	Definition of method(s) used to obtain reference standard	Sec 4.4, p.12	√	
	**15**	Rationale for choosing the reference standard	Sec 4.4, p.12	√	
	**16**	Source of reference standard annotations	Sec 4.4, p.12	√	
	**17**	Annotation of test set	Sec 4.4, p.12	√	
	**18**	Measures of inter- and intra-rater variability of features described by the annotators			√
Data Partitions	**19**	How data were assigned to partitions	Sec 4.4, p.12	√	
	**20**	Level at which partitions are disjoint	Sec 4.4, p.12	√	
Testing Data	**21**	Intended sample size	Sec 4.4, p.12	√	

Section / Topic	No.	Item	Page / Line	No	NA
Model	**22**	Detailed description of model	Sec 3.1-3.4, p.5-10	√	
	**23**	Software libraries, frameworks, and packages	Sec 4.2, p.11	√	
	**24**	Initialization of model parameters	Sec 4.3, p.11	√	
Training	**25**	Details of training approach	Sec 4.2, p.11; Sec 4.3, p.11	√	
	**26**	Method of selecting the final model	Sec 4.6, p.14	√	
	**27**	Ensembling techniques			√
Evaluation	**28**	Metrics of model performance	Sec 4.1, p.11	√	
	**29**	Statistical measures of significance and uncertainty	Sec 4.7, p.15-18 (Ablation studies)	√	
	**30**	Robustness or sensitivity analysis	Sec 4.9, p.18; Sec 5.2, p.20	√	
	**31**	Methods for explainability or interpretability	Sec 3, p.6-9	√	
	**32**	Evaluation on internal data	Sec 4.6, p.14-15; Sec 4.7, p.16	√	
	**33**	Testing on external data	Sec 4.9, p.18-19	√	
	**34**	Clinical trial registration			√
**RESULTS**					
Data	**35**	Numbers of patients or examinations included and excluded	Sec 4.4, p.12; Table 1	√	
	**36**	Demographic and clinical characteristics of cases in each partition			√
Model performance	**37**	Performance metrics and measures of statistical uncertainty	Sec 4.6, p.14-15; Sec 5, p.20	√	
	**38**	Estimates of diagnostic performance and their precision	Sec 4.6, p.14-15	√	
	**39**	Failure analysis of incorrect results	Sec 5.1, p.20 (Fig 21)	√	
**DISCUSSION**					

	**40**	Study limitations	Sec 5.2, p.20; Sec 7 (Conclusion)	√	
	**41**	Implications for practice, including intended use and/or clinical role	Sec 4.8, p.17-18;	√	
**OTHER INFORMATION**					
	**42**	Provide a reference to the full study protocol or to additional technical details	Data Availability Statement	√	
	**43**	Statement about the availability of software, trained model, and/or data	Data Availability Statement	√	
	**44**	Sources of funding and other support; role of funders	Funding Statement reads	√	

* Indicate page and/or line number for each checklist item that is present. NA = not applicable.
